# Functional Recovery After Surgical Treatment of Post-Burn Contractures: A Retrospective Cohort Study

**DOI:** 10.3390/healthcare14152381

**Published:** 2026-08-04

**Authors:** Zhanatay Ramaznov, Aliya Atepileva, Zhanar Akhmetkarimova, Serik Balgazarov, Yersultan Alzhanov, Aigul Ramazanova

**Affiliations:** 1 National Scientific Center of Traumatology and Orthopedics Named After Academician N.D. Batpenov, Abilaykhana Street 15, Astana 010000, Kazakhstan; 66zhanatay@mail.ru (Z.R.); balgazarov_s@niito.ru (S.B.); ersyltan@gmail.com (Y.A.); 2 National Center of Biotechnology, 13/5, Kurgalzhynskoye Road, Astana 010000, Kazakhstan; akhmekarimova@biocenter.kz; 3 Department of Dermatovenereology and Dermatocosmetology, Non-Profit Joint-Stock Company Astana Medical University, Beibytshilik Street 49, Astana 010000, Kazakhstan; agi_bota-97@mail.ru

**Keywords:** post-burn contracture, burn rehabilitation, functional recovery, scar maturation, reconstructive surgery, hand function, range of motion, Vancouver Scar Scale

## Abstract

**Highlights:**

**What are the main findings?**

- Scar maturation and functional recovery after post-burn contracture release did not always progress simultaneously.
- Persistent extension deficits were observed in a proportion of patients despite improvement in scar characteristics.

**What are the implications of the main findings?**

- Functional assessment may provide additional clinically relevant information beyond scar evaluation alone.
- Long-term rehabilitation and postoperative monitoring remain important after surgical correction of post-burn contractures.

**Abstract:**

Background: Surgical release of post-burn contractures aims to restore mobility and improve scar quality; however, the relationship between scar improvement and functional recovery remains incompletely understood, particularly in the hand. Methods: We performed a retrospective cohort study of 208 patients undergoing surgical correction of post-burn contractures (2021–2025) at a national referral center. Scar outcomes were assessed using the Vancouver Scar Scale (VSS). Functional outcomes were evaluated with joint-specific range-of-motion (ROM) measurements at 3, 6, and 12 months. For digital contractures, extension deficit was defined as ≥10° loss of active extension. Owing to non-normal distributions, data are presented as medians (IQR), and non-parametric tests were used. Results: VSS improved significantly across all domains (all *p* < 0.001). Median VSS remained stable between 3 and 6 months, followed by a marked decrease by 12 months. Functional recovery varied by region: major joints showed near-complete restoration of functional ROM, whereas extension deficit remained persistently prevalent, affecting 27.0% of patients at 3 months and 29.2% at 12 months. Full flexion was maintained in 88.8% of patients throughout follow-up. At the cohort level, flexion and extension demonstrated largely independent recovery trajectories. Conclusions: Surgical treatment improves scar characteristics; however, functional recovery—particularly extension in digital contractures—may remain incomplete. Combined assessment of scar characteristics and functional outcomes may provide a more comprehensive evaluation of postoperative recovery following contracture release.

## 1. Introduction

Post-burn contractures remain a significant cause of long-term disability following burn injury, leading to functional impairment, deformity, and reduced quality of life [1,2]. Surgical release is the mainstay of treatment for established contractures and aims to restore mobility while improving scar characteristics. A range of reconstructive techniques, including local tissue rearrangement and split-thickness skin grafting, are applied depending on anatomical location, severity, and available tissue [3].

Outcome assessment after contracture release has traditionally focused on scar-related parameters, most commonly evaluated using the Vancouver Scar Scale (VSS) [4]. While VSS provides a structured assessment of vascularity, pigmentation, pliability, and height, it primarily reflects morphological features of the scar rather than functional performance. Increasing evidence suggests that improvement in scar appearance does not necessarily translate into restoration of function, particularly in anatomically complex regions such as the hand [5].

Functional outcomes following burn reconstruction are less consistently reported and remain challenging to standardise. Range of motion (ROM) is commonly used as a surrogate marker of function; however, its clinical interpretation varies across joints [6,7,8].

In the hand, flexion and extension contribute differently to functional performance, including grasp, release, and fine motor tasks. Residual deficits—particularly in extension—may therefore have a disproportionate impact on overall hand function, even when scar characteristics appear satisfactory [9,10].

Clinical practice suggests that scar maturation and functional recovery do not always progress simultaneously after contracture release, particularly in patients with hand involvement. However, this relationship has not been adequately characterised in large clinical cohorts combining both scar and functional outcomes within the same population [11].

In particular, persistent extension deficit after surgical correction of digital contractures remains insufficiently described, despite its direct relevance to functional recovery and daily activities. The limited available data hinder the development of targeted surgical and rehabilitation strategies aimed at improving outcomes in this subgroup [12,13].

The present study aimed to evaluate scar and functional outcomes following surgical treatment of post-burn contractures in a large retrospective cohort. Special attention was given to patterns of functional recovery in digital contractures, with a focus on the prevalence, persistence, and clinical implications of extension deficit over time.

## 2. Methods

### 2.1. Study Design and Setting

This retrospective cohort study included patients who underwent surgical treatment for post-burn contractures between January 2021 and December 2025 at a national referral centre specialising in burn reconstruction. The centre receives patients from all administrative regions of the country, enabling analysis of a nationwide cohort with diverse clinical presentations.

### 2.2. Participants

All consecutive patients undergoing surgical correction of post-burn contractures during the study period were eligible. Inclusion criteria were: (1) documented burn injury, (2) presence of a functionally significant contracture requiring surgical release, and (3) availability of postoperative follow-up data for at least one time point.

Patients with missing key outcome variables were excluded from specific analyses but retained in descriptive analyses where appropriate.

All included patients had a history of deep dermal or full-thickness burns, inferred from the development of clinically significant contractures involving deeper soft tissues. Detailed data on burn depth and total body surface area (TBSA) were not consistently available due to the retrospective design. Therefore, the presence of clinically significant contractures requiring surgical correction was considered an indirect indicator of initially severe burn injury.

### 2.3. Surgical Procedures

The choice of surgical technique was based on anatomical location, type of contracture, and local tissue conditions. Two principal approaches were used: local tissue rearrangement (including Z-plasty and local flaps) and split-thickness skin grafting.

All procedures were performed after clinical scar maturation, defined by stabilisation of scar characteristics, reduced vascularity, decreased pliability, and absence of progressive contracture. In most cases, this corresponded to an interval of at least 6–12 months after the initial burn injury [12].

### 2.4. Postoperative Rehabilitation

A structured postoperative rehabilitation protocol was applied to all patients, with modifications according to anatomical location and type of reconstruction. Early controlled mobilisation was initiated as soon as clinically feasible, particularly following local tissue rearrangement, to reduce the risk of recurrent contracture [14].

For patients undergoing split-thickness skin grafting, a short period of immobilisation (typically 5–10 days) was used to facilitate graft take.

Rehabilitation for digital contractures included extension splinting, active and passive mobilisation, and physiotherapy-guided stretching. The intensity and duration of rehabilitation were individualised. Due to the retrospective design, adherence to rehabilitation protocols could not be fully quantified [14].

### 2.5. Outcome Measures

#### 2.5.1. Scar Assessment

Scar outcomes were assessed using the Vancouver Scar Scale (VSS) [15], evaluating vascularity, pigmentation, pliability, and height. Total VSS scores were recorded at 3, 6, and 12 months postoperatively.

#### 2.5.2. Functional Assessment

Functional outcomes were evaluated using joint-specific range-of-motion (ROM) measurements performed with standardised goniometry by trained clinicians [16]. For digital contractures, extension deficit was defined as a lack of active extension of ≥10° at the metacarpophalangeal or interphalangeal joints compared to the expected physiological range. The ≥10° threshold was adopted as a pragmatic institutional operational definition of clinically appreciable loss of active extension at the metacarpophalangeal and interphalangeal joints. We acknowledge that no universally validated goniometric criterion for clinically meaningful active extension deficit in post-burn digital contractures currently exists. Consistent with Parry et al., there is currently no consensus regarding what degree of contracture warrants specific clinical action, and no patient-anchored definition of clinical significance for loss of ROM in this setting [17]. The ≥10° cut-off used in the present analysis should therefore be interpreted as a descriptive institutional definition rather than as a validated clinical decision threshold. Both the presence and magnitude of extension deficit were recorded.

### 2.6. Data Collection

Demographic and clinical variables, including age, sex, burn aetiology, anatomical location, type of contracture, surgical technique and length of hospital stay, were extracted from institutional records.

Scar and functional data were analysed using partially overlapping datasets due to differences in documentation structure. Where possible, analyses were performed at the cohort level; however, direct patient-level linkage between scar and functional outcomes was not consistently available.

### 2.7. Statistical Analysis

Data normality was assessed using the Shapiro–Wilk test. As most variables were non-normally distributed, results are presented as medians with interquartile ranges (IQR), and non-parametric methods were used.

Paired comparisons over time were analysed using the Wilcoxon signed-rank test, while comparisons across multiple groups were performed using the Kruskal–Wallis test. Between-group comparisons were conducted using the Mann–Whitney U test or χ^2^ test, as appropriate.

Spearman’s rank correlation coefficient was used to assess associations between variables. Effect size (r) was calculated for paired comparisons.

A two-sided *p*-value < 0.05 was considered statistically significant. Statistical analyses were performed using Statistica 13.0 (TIBCO Software Inc., Palo Alto, CA, USA).

### 2.8. Ethics

The study was approved by the institutional ethics committee. Due to the retrospective design, the requirement for informed consent was waived.

## 3. Results

### 3.1. Baseline Characteristics

Between January 2021 and December 2025, a total of 233 patients underwent surgical treatment for post-burn contractures at a national referral centre (34 in 2021, 49 in 2022, 49 in 2023, 46 in 2024, and 55 in 2025). Of these, 208 met inclusion criterion 3 (availability of postoperative follow-up data for at least one time point) and comprised the analytical cohort; the remaining 25 patients (2025 cohort) were excluded due to insufficient follow-up duration at the time of data lock. Of the 208 included patients, 178 (cohorts 2021–2024) had completed 12-month follow-up and were used for longitudinal VSS and functional outcome analyses. The median age was 16 years (IQR 8–37), with children under 18 years comprising the majority of the cohort (57.2%, *n* = 119). Males slightly predominated (53.8%, *n* = 112).

Age distribution was as follows: <18 years −57.2% (*n* = 119), 18–40 years −21.2% (*n* = 44), 40–60 years −17.3% (*n* = 36), and >60 years −4.3% (*n* = 9). Annual case volume ranged from 34 to 55 patients across the full enrolment period. A significant increase in median age was observed over time, rising from 12 years in 2021–2022 to 30 years in 2025 (Kruskal–Wallis *p* < 0.001).

### 3.2. Geographic Distribution

Patients were referred from all 17 administrative regions of the country, reflecting the national coverage of the centre. The highest proportion originated from the Turkestan region (23.1%, *n* = 48), followed by Astana (10.1%, *n* = 21), Atyrau region (9.1%, *n* = 19), and Karaganda region (8.7%, *n* = 18). The top ten regions accounted for 73.1% of all cases.

### 3.3. Burn Etiology and Clinical Characteristics

Flame burns (45.7%, *n* = 95) and scald injuries (39.9%, *n* = 83) accounted for the majority of cases (85.6%). Contact burns were observed in 9.6% (*n* = 20), electrical injuries in 2.4% (*n* = 5), and other causes in 2.4% (*n* = 5).

Age-stratified analysis demonstrated distinct patterns: scald injuries predominated in children (55.7%), whereas flame burns were more common in adults (64.5%).

The most frequently affected anatomical site was the hand and fingers (42.3%, *n* = 88), followed by the wrist (12.0%), knee (11.5%), elbow (9.6%), foot and ankle (7.7%), shoulder (6.2%), and neck (5.8%).

### 3.4. Types of Contractures

Flexion contractures were the most common (56.7%, *n* = 118), followed by adduction contractures (15.4%, *n* = 32), extension contractures (13.5%, *n* = 28), and syndactyly (11.5%, *n* = 24).

Clear anatomical patterns were observed: flexion contractures predominated in the hand, wrist, knee, and elbow, whereas adduction contractures were mainly localised to the neck and shoulder. Syndactyly was confined to the hand.

The distribution of contracture types differed significantly across study years (χ^2^ = 30.64, df = 16, *p* = 0.015), largely due to the emergence of extension contractures in 2022. The baseline characteristics are summarized in Table 1.

### 3.5. Surgical Treatment

Primary procedures were performed in 94.7% of cases (*n* = 197), with only four patients (1.9%) undergoing reoperation. Z-plasty and local flap techniques were used in 54.8% (*n* = 114), while split-thickness skin grafting was performed in 45.2% (*n* = 94).

The choice of surgical method was strongly associated with contracture type (χ^2^ = 36.59, df = 4, *p* < 0.001): syndactyly was almost exclusively treated with Z-plasty (95.8%), whereas extension contractures were predominantly managed with skin grafting (85.7%). Flexion contractures were treated using both techniques in comparable proportions. A representative case illustrating the dissociation between scar maturation and functional recovery following split-thickness skin grafting is presented in Figure 1.

Skin grafting was associated with longer hospital stay compared with Z-plasty (median 11 [IQR 9–14] vs. 9 days [IQR 8–12]; *p* = 0.001). The overall median length of hospital stay was 10 days (IQR 8–13) (Figure 2).

The observed reoperation rate was low (1.9%). However, minor complications not requiring surgical revision may be underrepresented, and the 12-month follow-up may not capture late recurrence.

### 3.6. Scar Outcomes According to the Vancouver Scar Scale (VSS)

The distribution of total VSS scores at all time points deviated significantly from normality (Shapiro–Wilk test, *p* < 0.001); therefore, non-parametric methods were used.

Median total VSS decreased over time, from 6.0 (IQR 4.5–6.0) at 3 months to 6.0 (IQR 3.8–6.0) at 6 months and 1.0 (IQR 1.0–3.9) at 12 months. Pairwise comparisons demonstrated statistically significant differences between all time points (all *p* < 0.001).

Although median VSS remained unchanged between 3 and 6 months, the distribution of values differed significantly, indicating a shift in score distribution rather than stability of scar characteristics. The most pronounced reduction occurred between 6 and 12 months.

All VSS subscales improved significantly over time (all *p* < 0.001). Vascularity, pigmentation, and pliability approached near–normal values by 12 months, whereas scar height remained the most resistant component (median 1.0 across time points) (Figure 3).

Significant differences in VSS scores were observed across yearly cohorts at all time points (Kruskal–Wallis *p* < 0.001). Interannual VSS trajectories are presented in Table 2. The 2023 cohort demonstrated the lowest median VSS at 3 months (3.0) and reached a median of 0.0 by 6 months—the most rapid trajectory among all cohorts. In contrast, the 2022 cohort showed no significant change between 3 and 6 months (*p* = 0.126), with the main reduction occurring between 6 and 12 months.

### 3.7. Functional Outcomes

Functional outcomes were assessed using ROM and extension deficit in the cohort with complete 12-month follow-up (*n* = 178; cohorts 2021–2024). Restoration of ROM in major joints approached near-physiological ranges across follow-up, with no measurable extension deficit observed in these regions. In contrast, extension deficit remained persistently prevalent throughout the observation period: 48/178 (27.0%) at 3 months (median 12°), 49/178 (27.5%) at 6 months (median 15°), and 52/178 (29.2%) at 12 months (median 12°). The proportion of patients with extension deficit did not decrease over time; in two annual cohorts (2022 and 2024), a slight increase was observed at 12 months compared with 3 months. Extension deficit was confined to the hand, fingers, and wrist; no deficit was observed in other anatomical regions. Full flexion was achieved in 158/178 patients (88.8%) and remained stable across all time points. At the cohort level, flexion range did not demonstrate a clear relationship with extension deficit, suggesting that flexion and extension follow largely independent recovery trajectories. Notably, the persistence of extension deficit in the hand, wrist and digits was observed concurrently with progressive improvement in scar characteristics documented in Section 3.6, indicating that scar-based measures alone would not have captured this residual functional limitation.

### 3.8. Associations

Older age showed a weak but significant correlation with longer hospital stay (ρ = 0.205, *p* = 0.003). A representative case of staged paediatric lower limb reconstruction is presented in Figure 4.

Adults had longer hospitalisation compared with children (median 11 vs. 9 days, *p* = 0.003) (Figure 5).

No association was found between age and VSS improvement, and sex was not associated with surgical method, scar outcomes, or hospital stay.

## 4. Discussion

The present study demonstrated a dissociation between scar maturation and functional recovery following surgical correction of post-burn contractures [1].

While Vancouver Scar Scale (VSS) scores demonstrated progressive improvement across all domains, functional restoration—particularly active extension in digital contractures—remained incomplete in a substantial proportion of patients [6,7].

Scar maturation demonstrated a non-linear trajectory, with relative stability in median VSS values between 3 and 6 months, followed by a more pronounced reduction by 12 months [2]. This pattern likely reflects the biological phases of scar remodeling, where early structural changes (e.g., hypervascularity and metabolic activity) are not fully reflected in clinical scoring, while later phases are associated with evident improvements in pliability and color [8]. Notably, scar height remained the most resistant component, suggesting the persistence of deeper dermal alterations despite overall improvement in appearance [3,4].

A key observation was the persistence of extension deficit compared to major joints [6,7]. While large joints approached near-physiological ranges of motion, extension deficit remained present in 27–29% of patients across all follow-up time points, with no trend toward resolution at the cohort level [5,8]. This reflects the biomechanical complexity of the hand and wrist, where even minor residual stiffness in the extensor mechanism or periarticular tissues may significantly impair function [9]. The stability of extension deficit prevalence over 12 months—despite concurrent improvement in scar characteristics—suggests that postoperative recovery may depend not only on surgical correction, but also on prolonged rehabilitation and soft tissue adaptation [18].

The findings suggest that scar-related and functional recovery may progress along distinct trajectories following contracture release [1,5,10]. Full flexion was maintained in 88.8% of patients throughout follow-up, whereas extension deficit persisted in approximately one-quarter of the cohort without measurable improvement. Although range of motion is often reported as a single parameter, these findings indicate that flexion and extension follow largely independent recovery trajectories at the cohort level and should be evaluated separately in both clinical practice and research settings. This observation should be regarded as hypothesis-generating, as patient-level linkage between concurrent VSS and ROM data was not consistently available.

From a methodological perspective, the lack of detailed data on burn severity (TBSA) and depth represents a limitation inherent to the retrospective design [18]. However, the inclusion of patients requiring surgical contracture release allowed for the analysis of a clinically relevant cohort with advanced disease, using the need for surgical contracture release as a surrogate marker of injury severity [18]. The low reoperation rate (1.9%) suggests satisfactory early surgical outcomes, although it does not necessarily reflect complete functional recovery [19,20].

The persistence of extension deficit in approximately one-quarter of patients at 12 months warrants consideration of rehabilitation-related confounding. Our centre receives referrals from all 17 administrative regions of Kazakhstan, and the majority of patients are expected to have continued outpatient rehabilitation under regional supervision following discharge. The quality, intensity, and consistency of extension splinting and physiotherapy-guided mobilisation in regional facilities may vary substantially. While the intrinsic biomechanical resistance of the digital extensor mechanism and periarticular tissues likely contributes to the observed deficit persistence, the possibility that regional disparities in rehabilitation contribute to this outcome cannot be excluded with the available retrospective data. Future prospective studies with standardised rehabilitation tracking are needed to distinguish these factors [17].

From a clinical standpoint, these findings highlight the importance of extended follow-up and targeted rehabilitation strategies in patients with digital contractures [21]. The observed delay in both scar maturation and functional improvement suggests that the commonly used 6-month evaluation point may underestimate long-term recovery [19]. More fundamentally, the concurrent occurrence of progressive VSS improvement and persistent extension deficit at the same follow-up time points indicates that scar-based measures used in isolation may not capture the full spectrum of postoperative recovery, particularly in the hand and digits. Integration of structural (VSS) and functional (ROM) outcome measures therefore provides a more comprehensive assessment of treatment effectiveness in burn reconstruction, consistent with the conclusion of a systematic review of outcome tools in hand burns that no single instrument meets all psychometric properties of validity, reliability and responsiveness, and that a suite of measures spanning impairment to quality-of-life level is required [22].

### Strengths and Limitations

Several limitations should be considered when interpreting the findings of this study. The retrospective design resulted in variability in documentation completeness, particularly regarding initial burn severity and TBSA. Preoperative ROM values were not consistently documented and could not be included in the analysis, limiting assessment of the absolute magnitude of functional improvement. ROM was assessed using standard goniometry; recent work has highlighted that this method may have limited accuracy in the context of burn scar contracture assessment [17]. The reported prevalence figures should therefore be interpreted as approximations based on the measurement tool available within retrospective clinical practice. Scar and functional outcomes could not always be linked at the individual patient level; the observation that scar maturation and functional recovery follow distinct trajectories is therefore based on group-level data and should be regarded as hypothesis-generating. Furthermore, surgical technique and functional outcome data were recorded as separate aggregate variables, precluding patient-level subgroup analysis of extension deficit by operative approach. Rehabilitation adherence varied between patients and could not be fully quantified; the possibility that regional disparities in outpatient rehabilitation—including inconsistent use of extension splinting and physiotherapy-guided mobilisation—contribute to the persistence of extension deficit cannot be excluded and warrants investigation in future prospective studies with standardised rehabilitation tracking. Scar maturation was assessed using clinical criteria rather than quantitative measurements. Follow-up was limited to 12 months, which restricts assessment of long-term recurrence and late functional outcomes.

## 5. Conclusions

Surgical release of post-burn contractures is associated with substantial improvement in scar characteristics. In the present cohort, however, functional recovery followed a distinct trajectory: while VSS scores continued to improve through 12 months, active extension deficit in the hand, wrist, and digits persisted in 27–29% of patients throughout follow-up, whereas full flexion was maintained in 88.8% of cases and major-joint ROM approached physiological values. These observations indicate that scar-based measures interpreted in isolation may not capture the full spectrum of postoperative recovery, particularly in digital and hand contractures.

Building on this dissociation, we propose that postoperative follow-up after surgical correction of post-burn contractures should adopt a dual-domain assessment framework. In this approach, validated scar-based measures (such as the VSS) are complemented by joint-specific active ROM assessments, with particular attention to active extension deficits in the hand and digits. This proposal aligns with a systematic review of outcome tools in hand burns, which established that no single outcome measure satisfies all psychometric properties of validity, reliability, and responsiveness, and that a comprehensive evaluation of post-burn hand recovery requires a suite of tools spanning impairment, activity, and quality-of-life domains [20].

We do not propose a specific numerical threshold for active extension deficit at this stage. Standard goniometric assessment in the post-burn setting has itself been called into question, with reported underestimation of ROM impairment and no current consensus regarding what degree of contracture warrants clinical action [17]. Establishing validated, joint-specific functional benchmarks—including a reliable definition of clinically meaningful active extension deficit—should therefore be a priority for future prospective, patient-level–linked studies. In the interim, combined interpretation of scar and functional outcomes at each follow-up visit provides a more comprehensive assessment of reconstructive results than reliance on either domain alone.

## Figures and Tables

**Figure 1:**
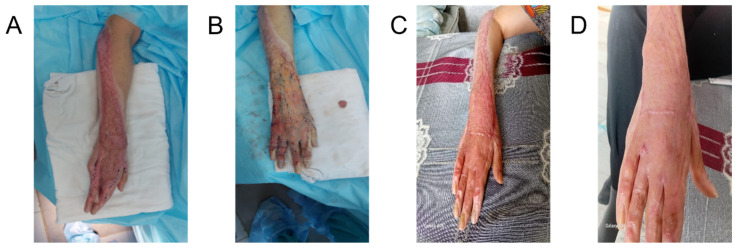
Representative case of post-burn hand contracture demonstrating dissociation between scar maturation and functional recovery. (**A**) Preoperative appearance showing severe flexion–extension contractures of the wrist and digits. (**B**) Early postoperative result following surgical release and split-thickness skin grafting. (**C**) Six-month follow-up demonstrating partial scar maturation with persistent functional limitation. (**D**) Twelve-month follow-up showing satisfactory scar maturation; however, extension deficit in the proximal interphalangeal joints remains evident.

**Figure 2:**
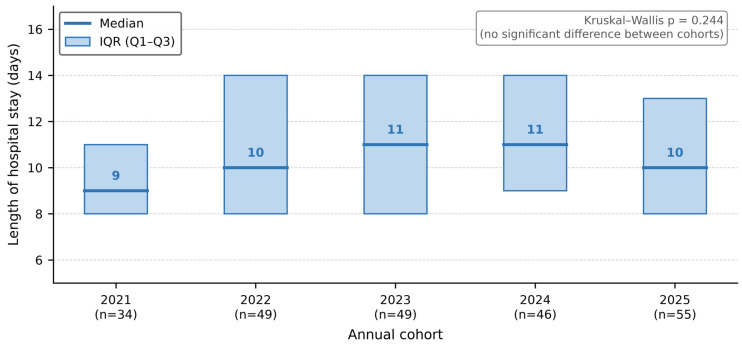
Length of hospital stay by annual cohort (*n* = 233). Box plots show median and interquartile range for each enrolment year. No significant difference was observed between cohorts (Kruskal–Wallis *p* = 0.244).

**Figure 3:**
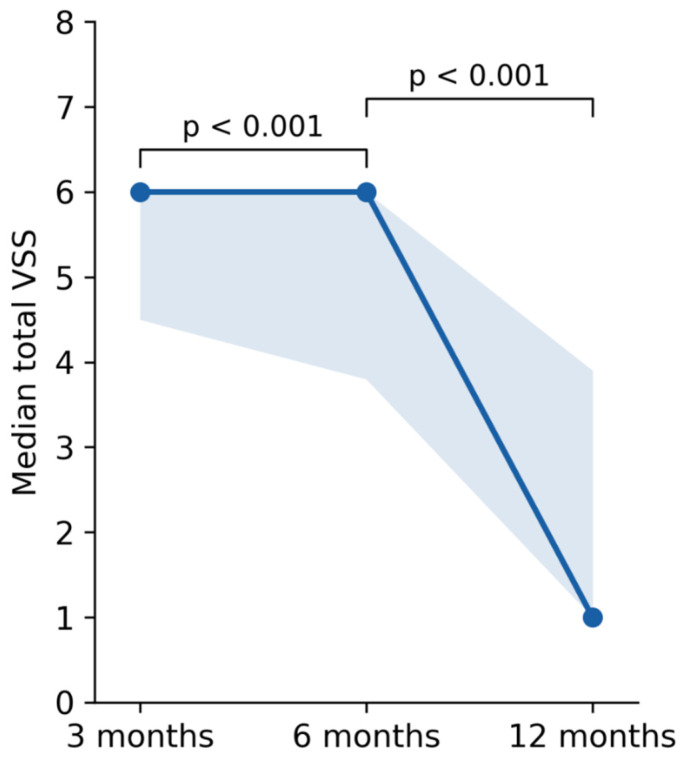
Changes in total VSS over time (*n* = 178). The line represents the median values, and the shaded area represents the interquartile range (IQR, Q1–Q3). Pairwise comparisons were performed using the Wilcoxon signed-rank test.

**Figure 4:**
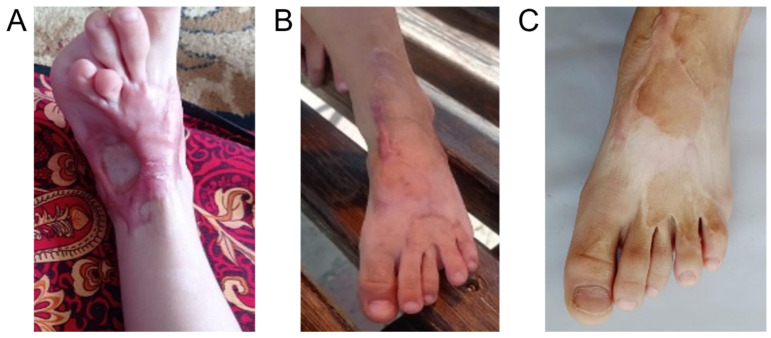
Longitudinal outcome of staged reconstruction of lower limb contracture in a paediatric patient. (**A**) Preoperative appearance demonstrating severe post-burn contracture of the ankle and foot with digital deformities. (**B**) Six-month follow-up showing substantial improvement in ankle alignment and range of motion. (**C**) Twelve-month follow-up demonstrating satisfactory restoration of ankle function; however, residual deformities of the toes remain evident.

**Figure 5:**
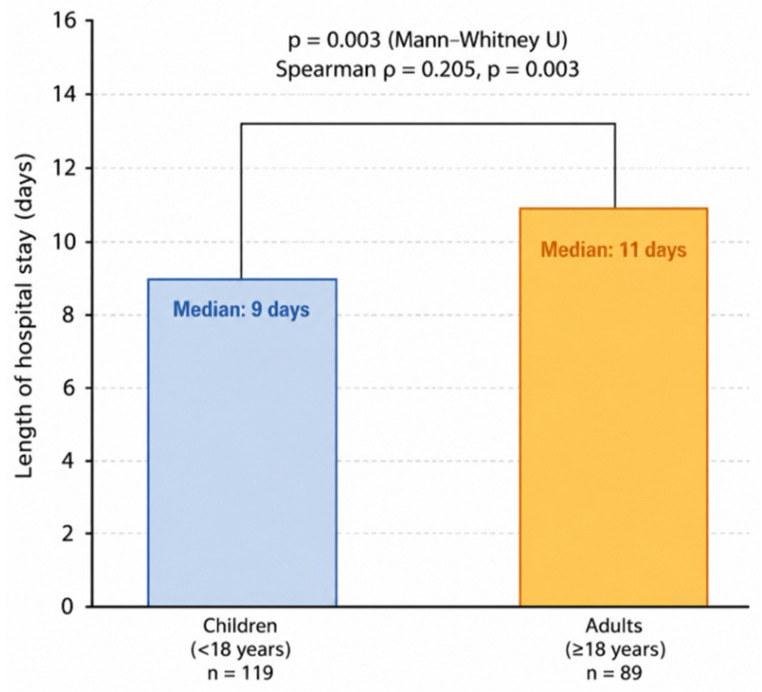
Length of hospital stay by age group. Children (<18 years, *n* = 119, median 9 days) vs. adults (≥18 years, *n* = 89, median 11 days); Mann–Whitney U *p* = 0.003; Spearman ρ = 0.205, *p* = 0.003.

**Table 1:** Patient characteristics.

Variable	*n* (%)
Demographics	
Total patients	208
<18 years	119 (57.2)
18–40 years	44 (21.2)
40–60 years	36 (17.3)
>60 years	9 (4.3)
Male sex	112 (53.8)
Burn etiology	
Flame	95 (45.7)
Scald	83 (39.9)
Contact	20 (9.6)
Electrical	5 (2.4)
Other causes	5 (2.4)
Type of contracture	
Flexion	118 (56.7)
Adduction	32 (15.4)
Extension	28 (13.5)
Syndactyly	24 (11.5)
Mixed	4 (1.9)
Anatomical location	
Hand and fingers	88 (42.3)
Wrist	25 (12.0)
Knee	24 (11.5)
Elbow	20 (9.6)
Foot and ankle	16 (7.7)
Shoulder	13 (6.2)
Neck	12 (5.8)
Hip joint	10 (4.8)

**Table 2:** VSS scores by annual cohort (cohorts 2021–2024, *n* = 178).

Cohort	VSS 3 mo, Median (IQR)	VSS 6 mo, Median (IQR)	VSS 12 mo, Median (IQR)	*p* (3→6 mo)	*p* (3→12 mo)
2021 (*n* = 34)	7.0 (7.0–7.0)	4.0 (3.8–6.0)	1.0 (0.0–1.2)	<0.001	<0.001
2022 (*n* = 49)	6.0 (5.8–6.0)	6.0 (5.9–6.0)	4.0 (3.9–4.0)	0.126	<0.001
2023 (*n* = 49)	3.0 (3.0–6.0)	0.0 (0.0–6.0)	0.0 (0.0–1.6)	<0.001	<0.001
2024 (*n* = 46)	6.0 (6.0–6.0)	6.0 (6.0–6.0)	1.0 (1.0–1.0)	not significant.	<0.001
Pooled (*n* = 178)	6.0 (4.5–6.0)	6.0 (3.8–6.0)	1.0 (1.0–3.9)	<0.001	<0.001
KW *p* (between cohorts)	<0.001	<0.001	<0.001	—	—

## Data Availability

The study is based on retrospective clinical data obtained from patient medical records at a national referral center. Due to ethical and privacy considerations related to patient confidentiality, the full dataset cannot be made publicly available. Therefore, we selected the “Dataset available on request from the authors” option. De-identified data may be provided by the corresponding author upon reasonable request and subject to institutional and ethical regulations.
